# Iatrogenic Obturator Hip Dislocation with Intrapelvic Migration

**DOI:** 10.1155/2018/5072846

**Published:** 2018-07-09

**Authors:** Shachar Kenan, Spencer Stein, Robert Trasolini, Daniel Kiridly, Bruce A. Seideman

**Affiliations:** ^1^Department of Orthopaedics, Hofstra North Shore Long Island Jewish, Northwell Health Medical Center, New Hyde Park, NY, USA; ^2^Department of Orthopaedics, New England Baptist Hospital, Boston, MA, USA; ^3^Department of Orthopaedics, Hofstra North Shore Long Island Jewish, Northwell Health Medical Center, St. Francis Hospital, Roslyn, NY, USA

## Abstract

Obturator hip dislocations are rare, typically resulting from high-energy trauma in native hips. These types of dislocations are treated with closed reduction under sedation. Open reduction and internal fixation may be performed in the presence of associated fractures. Still rarer are obturator hip dislocations that penetrate through the obturator foramen itself. These types of dislocations have only been reported three other times in the literature, all within native hips. To date, there have been no reports of foraminal obturator dislocations after total hip arthroplasty. We report of the first periprosthetic foraminal obturator hip dislocation, which was caused iatrogenically during attempts at closed reduction of a posterior hip dislocation in the setting of a chronic greater trochanter fracture. Altered joint biomechanics stemming from a weak hip abductor mechanism rendered the patient vulnerable to this specific dislocation subtype, which ultimately required open surgical intervention. An early assessment and identification of this dislocation prevented excessive closed reduction maneuvers, which otherwise could have had detrimental consequences including damage to vital intrapelvic structures. This case report raises awareness to this very rare, yet potential complication after total hip arthroplasty.

## 1. Introduction

Total hip arthroplasty (THA) has been the treatment of choice for patients with end stage femoroacetabular joint degeneration with the goals of relieving pain, restoring function, and improving quality of life. Possible complications include infection, neurovascular damage, dislocation, periprosthetic fracture, aseptic loosening, and leg length discrepancy. Dislocation, one of the most common complications after THA, occurs in approximately 0.3% to 10% of primary THAs and up to 28% for revision THA [1–9]. A meta-analysis of 260 clinical studies, which included 13,203 primary total hip arthroplasties, noted dislocation rates of 3.23%, 2.18%, and 0.55% for posterior, anterolateral, and direct lateral approaches, respectively [10]. Patient risk factors include older age, female gender, prior surgery, neuromuscular disorders, dementia, and alcohol abuse [11]. Surgical risk factors include component malpositioning, failure to restore leg length or offset, posterior approach, and implants which decrease the head to neck ratio [11]. Anatomically, hip dislocations are described as anterior or posterior to the acetabulum. Anterior hip dislocations are further subclassified as superior, inferior, luxation erecta of hip, obturator, or pubic type [12].

Inferior obturator dislocations tend to be traumatic, occurring with hip flexion, external rotation, and forced abduction. Due to the rarity of this type of dislocation, it is difficult to assess its true incidence. To our knowledge, only 29 cases of obturator hip dislocation have been reported in the literature [12–36]. Dislocations of this nature typically occurred in native hips in the setting of trauma, with a majority being associated with femoral neck, head, or acetabular fractures. Three of these documented dislocations described the displacement of the femoral head with penetration through the obturator foramen; however, those cases were all within native hips, and none were periprosthetic [13, 14, 18].

This case report is the first documented description of a periprosthetic foraminal obturator hip dislocation. The patient is an 83-year-old female, sixteen years status post right posterior total hip arthroplasty, who sustained an iatrogenic obturator hip dislocation with femoral head component penetration through the obturator canal resulting from an attempt at closed reduction of a posterior hip dislocation. The authors have obtained the patient's informed written consent for print and electronic publication of the case report.

## 2. Case Presentation

### 2.1. Clinical

**Figure 1:**
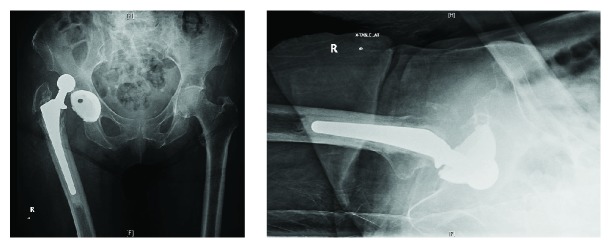
Anteroposterior (AP) pelvis and lateral right hip radiographs showing a posterosuperior dislocation of the right cemented femoral component with associated chronic greater trochanteric periprosthetic fracture and chronic left inferior pubic rami fracture.

**Figure 2:**
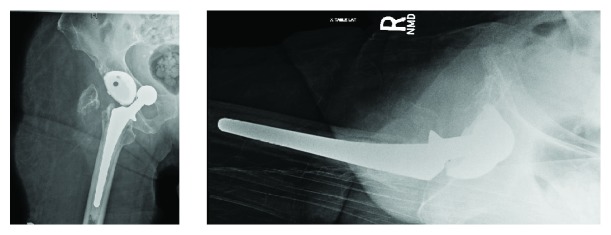
Anteroposterior (AP) and lateral right hip radiographs, status post attempted closed reduction revealing right iatrogenic obturator hip dislocation with femoral component intrapelvic migration.

**Figure 3:**
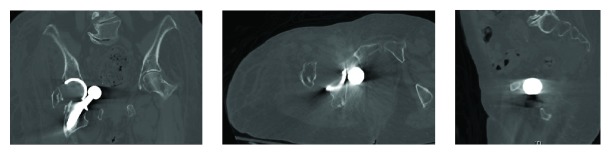
Coronal, axial, and sagittal computed tomography (CT) images showing femoral component dislocation through the right obturator canal and abutting the urinary bladder.

**Figure 4:**
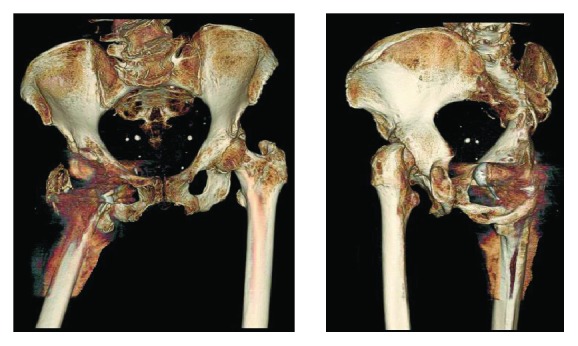
Three-dimensional reformatted computed tomography (CT) images showing femoral component dislocation through the right obturator canal.

An 83-year-old female with a past medical history of rheumatoid arthritis (on DMARD's), asthma, depression, gastroesophageal reflux disease (GERD), and lumbar spondylosis, as well as a past surgical history of right posterior total hip arthroplasty (1999), bilateral total knee arthroplasties (2003, 2012), and right shoulder hemiarthroplasty (2010), presented with five days of right hip pain and inability to ambulate after bending down. In the emergency department, initial radiographs revealed a right posterior hip dislocation, as well as chronic appearing fractures of the right greater trochanter and left inferior public rami (Figure 1). Her right lower extremity was shortened, internally rotated, and adducted. A propofol-induced conscious sedation was performed by the emergency physician and closed reduction was attempted by an experienced orthopaedic resident. The reduction maneuver involved hip flexion, traction, adduction, and internal rotation followed by external rotation and abduction. After three attempts, post reduction radiographs were significant for a right inferior obturator hip dislocation (Figure 2). The patient tolerated the procedure and was neurovascularly intact distal to her hip. Computed tomography (CT) was performed, which confirmed a persistently dislocated femoral head with intrapelvic migration through the right obturator foramen (Figures 3 and 4). Having failed three attempts at closed reduction, the patient was taken to the operating room for open reduction and revision arthroplasty.

**Figure 5:**
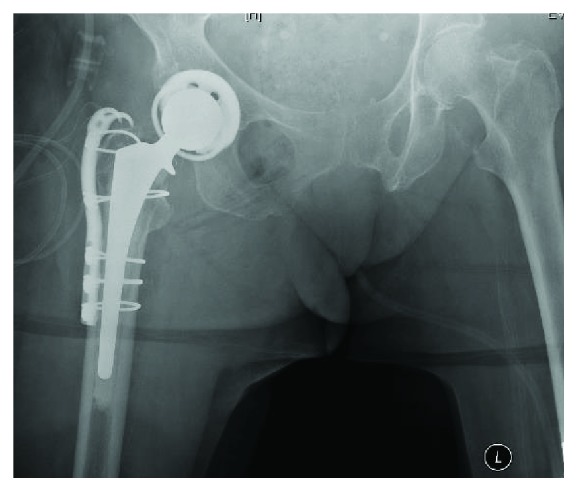
Anteroposterior (AP) pelvis radiograph, status post right hip open reduction, revision total hip arthroplasty with constrained liner and greater trochanteric hook plate with cerclage cables.

Using a posterolateral approach, the femoral head was found to be locked inferior and posterior to the acetabulum. Manual traction was utilized to successfully extricate the femoral component from within the obturator ring. Both the femoral and acetabular components were stable; however, a large amount of posterior wear was noted on the liner, which was exchanged for a constrained component. A greater trochanteric hook plate with cerclage cables was then utilized for the fixation of the greater trochanteric fragment (Figure 5). Excellent stability with a full range of motion was noted.

**Figure 6:**
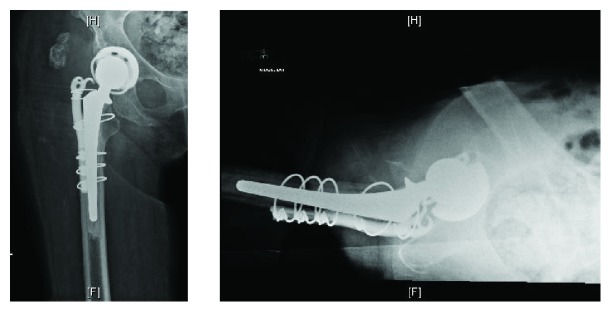
Anteroposterior (AP) and lateral right hip radiographs, seven months status post revision total hip arthroplasty with greater trochanter escape from hook plate.

**Figure 7:**
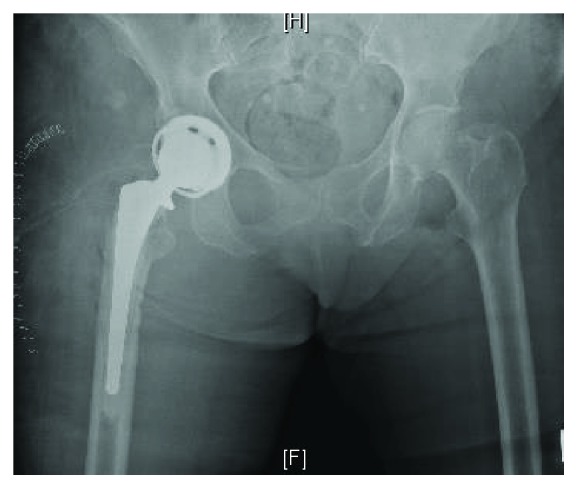
Anteroposterior (AP) pelvis radiograph, status post right hip irrigation and debridement, greater trochanter excision, hook plate, and cable removal of hardware.

Postoperatively, the patient was weight bearing as tolerated, with standard posterior hip precautions including an abduction pillow. Aspirin 325 mg BID was used for deep vein thrombosis (DVT) prophylaxis. Although the patient initially did very well, she developed urosepsis six months after the index procedure, leading to an acute right periprosthetic septic hip with *Proteus mirabilis*. Radiographs showed greater trochanteric escape from the hook plate (Figure 6). She then underwent irrigation and debridement with greater trochanter excision and hook plate removal (Figure 7). The patient was discharged with 6 weeks of ceftriaxone antibiotics via a peripherally inserted central catheter and has since been doing well with no further dislocations.

## 3. Discussion

Obturator hip dislocation after total hip arthroplasty is a rare complication. The nature of dislocation is dependent on a multitude of factors, with trauma being the most common predisposing factor. In the setting of trauma, patients may present with associated injuries such as external iliac artery occlusion, ipsilateral fractures of the acetabulum, femoral neck, greater trochanter, or femoral shaft, as well as long-term sequelae such as myositis ossificans [37]. Unlike periprosthetic hips, native hip dislocations may additionally present with femoral head impaction fractures resulting from impaction of the femoral head on the anteroinferior rim of the acetabulum [37]. Such impaction fractures lead to femoral head defects, similar to Hill-Sachs lesions of the proximal humerus after anterior shoulder dislocations.

We described an iatrogenic obturator anterior hip dislocation in a patient who had sustained a subacute posterior hip dislocation in association with a chronic greater trochanteric fracture. The patient was treated with revision arthroplasty and greater trochanteric open reduction internal fixation (ORIF). A fracture of the greater trochanter after total hip arthroplasty is classified as a Vancouver AG periprosthetic fracture [38]. According to a study of 32,644 primary total hip arthroplasties, a Vancouver AG fracture was the most common subtype of fracture, occurring in 32% of patients who sustained a postoperative periprosthetic hip fracture [39]. The overall rate of periprosthetic hip fractures was 3.5% in this same study group. The treatment of these fractures depends on the amount of displacement. For minimally displaced Vancouver AG fractures, patients are treated conservatively, with protected weight bearing and abductor hip precautions [40]. Displaced greater trochanter fractures require surgical fixation using wires, screws, cables, or specialized plates [40]. In our case, ORIF was performed due to the associated hip dislocation and fragment instability.

There is a paucity of literature describing obturator anterior hip dislocations after total hip arthroplasty. Most cases report native hip obturator dislocation following significant trauma with only three confirmed cases of femoral head penetration through the obturator foramen. These patients included a 24-year-old female with Ehlers-Danlos syndrome, a 33-year-old who presented with a neglected obturator dislocation six months after injury, and a 40-year-old female after a horse riding accident [13, 14, 18].

We believe that our patient's subacute presentation coupled with a preexisting greater trochanteric fracture contributed to an obturator hip dislocation after standard hip reduction attempts. Decreased abductor forces due to the greater trochanteric fracture led to hip instability, allowing the femoral prosthesis to migrate anteriorly and inferiorly. Post reduction three-dimensional reformatted CT scans (Figure 4) excellently illustrate this rare anatomic deformity.

## 4. Conclusion

This case serves as an example of anterior obturator hip dislocation after an attempt at closed reduction. It is important to understand that the mechanism of abduction and external rotation resulting in obturator hip dislocation is the same maneuver that is used during standard hip dislocation reduction attempts. Great care should therefore be taken when attempting a closed reduction in the presence of an ipsilateral greater trochanteric fracture, with radiographs performed after each attempt. Multiple failed attempts in this setting may eventually lead to incarceration of the femoral head through the obturator foramen, which should be confirmed by radiographs and computed tomographic (CT) scans.

In the setting of a confirmed foraminal obturator hip dislocation, there should be a low threshold for open reduction to avoid damage to neighboring critical intrapelvic structures from excessive closed reduction attempts. Furthermore, this case highlights the importance of close follow-up, especially in patients who are immunosuppressed and are at a high risk of periprosthetic infection. Early detection and treatment of potential sources of infection such as open wounds and ulcers, urinary tract infections (UTIs), and respiratory infections are critical to preventing hematogenous spread. Awareness of patient-specific factors that alter hip biomechanics, such as abductor mechanism disruption, should prompt added care and precaution during traditional closed reduction maneuvers, helping the treating orthopaedist avoid this type of dislocation.
